# Cross-Feature Hybrid Associative Priori Network for Pulsar Candidate Screening

**DOI:** 10.3390/s25133963

**Published:** 2025-06-26

**Authors:** Wei Luo, Xiaoyao Xie, Jiatao Jiang, Linyong Zhou, Zhijun Hu

**Affiliations:** 1 School of Computer Science and Technology, Guizhou University, Huaxi Avenue, Huaxi District, Guiyang 550025, China; wluo@gzu.edu.cn; 2 Key Laboratory of Information and Computing Science of Guizhou Province, Guizhou Normal University, Baoshan North Road, Yunyan District, Guiyang 550001, China; jjt@gznu.edu.cn (J.J.); gzmtzly@126.com (L.Z.); 3 School of Mathematics and Statistics, Guangxi Normal University, Guilin 541004, China; huzhijun@mailbox.gxnu.edu.cn

**Keywords:** pulsar candidates, deep learning, FAST, hybrid association, cross-feature fusion, radio astronomy

## Abstract

To enhance pulsar candidate recognition performance and improve model generalization, this paper proposes the cross-feature hybrid associative prior network (CFHAPNet). CFHAPNet incorporates a novel architecture and strategies to integrate multi-class heterogeneous feature subimages from each candidate into multi-channel data processing. By implementing cross-attention mechanisms and other enhancements for multi-view feature interactions, the model significantly strengthens its ability to capture fine-grained image texture details and weak prior semantic information. Through comparative analysis of feature weight similarity between subimages and average fusion weights, CFHAPNet efficiently identifies and filters genuine pulsar signals from candidate images collected across astronomical observatories. Additionally, refinements to the original loss function enhance convergence, further improving recognition accuracy and stability. To validate CFHAPNet’s efficacy, we compare its performance against several state-of-the-art methods on diverse datasets. The results demonstrate that under similar data scales, our approach achieves superior recognition performance. Notably, on the FAST dataset, the accuracy, recall, and F1-score reach 97.5%, 98.4%, and 98.0%, respectively. Ablation studies further reveal that the proposed enhancements improve overall recognition performance by approximately 5.6% compared to the original architecture, achieving an optimal balance between recognition precision and computational efficiency. These improvements make CFHAPNet a strong candidate for future large-scale pulsar surveys using new sensor systems.

## 1. Introduction

Pulsars are neutron stars with strong gravitational and magnetic fields, extreme density and rapid rotation [1]. Studying pulsars can help us better understand the nature of the universe and matter. For example, testing the predictions of general relativity requires observing the rotation of pulsars with high precision, such as gravitational redshift and light deflection, as well as others [2]. The properties and distribution of pulsars can be used to study the evolution of galaxies, the structure of the Milky Way, and the properties of interstellar media, etc. [3]. Pulsars can be used to explore the existence and properties of gravitational waves by measuring their rotation period, rotation speed, and other parameters [4].

In large-scale radio astronomy surveys, various sensors in radio telescopes play a key role in data collection. They continuously gather a huge amount of radio wave signals from space. These signals are then converted into data forms such as pulsar candidate images. Some of these surveys are the Parkes survey (PMPS) [5], the High Time Resolution Universe (HTRU) survey [6], the Pulsar Arecibo Lband Feed Array (PALFA) survey [7], the Green Bank Telescope (GBT) drift-scan pulsar survey [8], the Green Bank North Celestial Cap (GBNCC) survey [9], and the Low-Frequency Array (LOFAR) Tied-Array All-Sky Survey (LOTAAS) [10]. However, only a small fraction of the large number of potential candidates generated in modern pulsar surveys are confirmed to be genuine pulsars. For example, the new generation Five-hundred-meter Aperture Spherical Telescope (FAST) [11] is equipped with a fast 19-beam receiver, produces more than 1 million pulsar candidate stars every night and is expected to discover 5000 pulsars. According to conservative estimates (based on the sample ratio of pulsars/non-pulsars in HTRU data: 1/10,000 [12]), the Square Kilometer Array (SKA) [13] will find 20,000 pulsars from the expected discovery of 200 million samples. Taking FAST as an example, after the device receives periodic signals, we will utilize search software such as the PulsaR Exploration and Search Toolkit (PRESTO) [14,15] to perform a series of data processing. After dispersion and period folding processing, possible combinations of pulsar rotation parameters and statistical distribution results are obtained, which are called pulsar candidates [16,17,18]. The processed data are converted into image form for identifying the main features of pulsars.

These candidate samples will be further classified and screened to select valuable pulsar candidate signals for observational confirmation, a process known as pulsar candidate sample classification. The goal of classification is to reduce the retention of non-pulsar signals, thereby minimizing the workload for further observations while maximizing the chances of retaining pulsar signals. However, accurately and effectively filtering valuable pulsar candidate samples from vast amounts of data for further observation and confirmation is an important problem that needs to be addressed [19,20].

Traditional pulsar recognition methods mainly rely on manual feature extraction and shallow machine learning models. For example, the PICS framework proposed by Zhu et al. (2014) enhances classification robustness through feature interaction, but its high computational complexity makes it difficult to process large-scale survey data [21]. In contrast, Lyon et al. (2016) used a specially constructed tree-based machine learning classifier, the Gaussian Hellinger Very Fast Decision Tree, along with a set of new features to describe candidates, thereby quickly selecting promising candidates [22]. However, while these methods improved screening efficiency to some extent, their feature extraction process was highly dependent on expert experience, making it difficult to handle complex and variable noise environments and limiting their generalization ability for low signal-to-noise ratio (SNR) candidates [23,24,25]. Wang et al. (2019) used tree-based models to select candidate features and employed random forests, XGBoost, and hybrid ensemble methods to classify imbalanced pulsar candidates. By correcting the chi-square value of the pulse profile and the signal-to-noise ratio (SNR), they were able to identify genuine pulsar images [26]. Guo et al. (2019) proposed the DCG-AN-L2-SVM framework, which combines deep features with support vector machines to address data imbalance and enhance the model’s generalization ability [27].

In recent years, deep learning has made significant progress and had profound impacts, greatly influencing research in the field of astronomy [28]. With the resurgence of neural networks, especially convolutional neural networks (ConvNets), the field of visual recognition has successfully shifted from engineered features to the design of ConvNet architectures [29]. Slabbert et al. (2024) verified the feasibility of combining classical artificial neural networks (CNNs) with quantum neural networks (QNNs) for pulsar recognition [30]. However, CNNs tend to overlook global context information, leading to insufficient modeling of the overall shape of pulse profiles [31,32,33].

With Dosovitskiy et al. (2020) introducing the Vision Transformer (ViT) [34], researchers have found that with the help of larger models and dataset sizes, ViT demonstrates significantly better recognition performance [35,36,37,38,39]. The Transformer is a milestone work in this direction, achieving approximate global attention (global feature extraction) through window-based self-attention and sliding window self-attention. It has been widely applied in natural language processing [40,41] and computer vision fields [42,43,44]. As a universal visual backbone, SwinTransformer [45] excels at long-range dependency modeling through its attention mechanism. This capability facilitates cross-scale semantic association, making it naturally suited for fusing information from diverse modalities or views [46,47]. It provides an effective mechanism for multi-modal reasoning [48,49,50] and has achieved state-of-the-art performance in a range of computer vision tasks. For example, Wang et al. [39] improved performance and efficiency by introducing overlapping patch embedding, convolutional feed-forward networks, and linear attention through PVTv2. Prakash et al. [47] proposed using Transformers to integrate attention across image and LiDAR representations. In addition, the parallelization capabilities of Swin Transformer, the expressive power of its attention mechanism, and its relatively stable training process demonstrate strong scalability in terms of model size and data volume. It is highly suitable for processing large-scale pulsar data.

We apply the Swin Transformer for pulsar recognition, as well as for track changes in attention weights and fusion features. This helps us find logical connections between different subimages, just like what human experts do. We also look for ways to extract more graphic details and improve the loss function, making our network more accurate and stable.

The main contributions of this article are summarized as follows:

- A cross-feature hybrid associative prior network (CFHAPNet) and corresponding strategies are proposed. The method integrates multi-channel data composed of heterogeneous subimages from each pulsar candidate through hybrid association. By tracking, fusing, and comparing feature weight mappings across channels, it effectively addresses the challenge of associating relatively independent multi-view subimages of pulsar candidates for recognition.
- Structural enhancements are implemented in the encoder through adjustments to the downsampling scheme, the addition of optional layers, and the introduction of a cross-feature fusion (CFF) module. These modifications improve the model’s capacity to capture fine-grained textural details in multi-view subimages and refine the generation of weak semantic priors, thereby achieving significant improvements in recognition accuracy for candidate subimages.
- The loss function is enhanced through the introduction of cosine loss and learnable parameters, which aim to strengthen intra-class cohesion and inter-class separability. These refinements address the original loss function’s limitations in effectively discriminating hard samples and handling feature ambiguity.

The rest of this article is organized as follows. In Section 2, we provide a detailed description of the proposed model, introducing the improvements to the encode module. We also discuss in detail how to enhance the loss function. In Section 3, we introduce the experimental datasets, training parameters, and evaluation metrics. In Section 4, we utilize many experiments to verify the superiority of our method. In Section 5, the results of each experiment are discussed. In Section 6, the proposed method is summarized.

## 2. Proposed Methods

**Figure 1:**
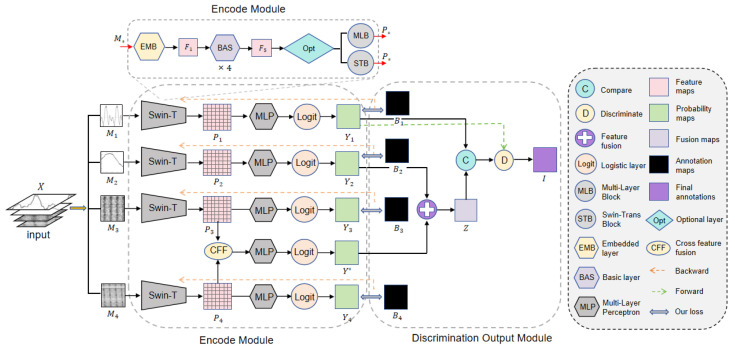
Overall structure of cross-feature hybrid associative prior network.

Because pulsar signals are weak and often interfered with by terrestrial signals, it is difficult to identify genuine pulsar images based on just one type of candidate subimage. Human experts commonly use four different types of candidate subimages, namely average pulsar profile plot, DM curve plot, time–phase plot, and frequency–phase plot, to correlate and identify pulsars. However, mimicking the way human experts recognize pulsars by automatically correlating these heterogeneous subimages is one of the challenges in the field of automatic pulsar recognition. Traditional methods of recognizing candidates by simple multi-subimage mosaicking have achieved preliminary automation, but due to the large image size and the need to extract multiple heterogeneous features simultaneously, it is hard to balance recognition accuracy and efficiency. The cross-feature hybrid associative prior network (CFHAPNet) we propose takes these four types of subimages as inputs and establishes effective logical correlations between them by tracking, fusing, and comparing the recognition weights of each subimage, thus efficiently screening out genuine pulsar candidates. The detailed structure of CFHAPNet is given in Figure 1.

*X* represents the set of candidate images, which is composed of multi-channel data from four different types of candidate subgraphs. *X* is fed into the weight tracking module in the order of the average pulsar profile, DM curve, time–phase diagram, and frequency–phase diagram. This structure effectively ranks the four subgraphs in a sequence that reduces the recall rate and improves precision, forming a mechanism similar to a funnel-shaped hierarchical filtering process. The initial image has a higher recall rate to ensure that the initial subgraph recognition results contain as many real pulsar signals as possible, minimizing the likelihood of missing any pulsar signals. As the recognition accuracy improves for each subsequent subgraph, pulsar signals are progressively identified correctly, while interference signals are eliminated.

The weight tracking, fusion and output processes form a hybrid associative architecture through combined serial–parallel connections, implemented progressively in two consecutive stages: First, during the encoding phase, the original label maps of four distinct subimage categories serve as learning targets for training the encoding module, producing corresponding prior weight maps $Y_{i}\left(i=1,2,3,4\right)$ for each subimage $M_{i}$. A cross-feature fusion (CFF) module is introduced to produce the cross-feature fusion weight $Y^{*}$.

Subsequently, in the discriminative output module, $Y_{2}$ and $Y^{*}$ are fused in parallel to generate the weight fusion map *Z*. The internal weight distances within $Y_{1}$ and *Z* are then separately calculated and compared to analyze weight variation trends. Based on these trend variations combined with $Y_{1}$, a discriminative weighting process is applied to generate the final result set *I*.

The detailed process will be elaborated in the following subsections:

### 2.1. Encode Module

In the encode module, the encoder adopts a network architecture based on the Swin Transformer for generating features for each type of image. The Swin Transformer model employs a hierarchical construction method commonly used in convolutional neural networks, i.e., patch merging, for hierarchical feature extraction through image reconstruction. Its window-based self-attention computation strategy reduces the computational complexity to a linear scale of the image size. This approach offers advantages in terms of consuming computational resources and improving accuracy when dealing with large datasets such as pulsar data.

#### 2.1.1. Optional Layer

Due to the relatively blurred signal boundaries in the time–phase and frequency–phase images of certain pulsar candidate subgraphs, oversized initial patches may diminish the encoder’s ability to capture fine-grained texture information. We reduced the initial patch size from Swin Transformer’s default 4 × 4 pixels to 2 × 2 pixels. However, with the new initial patch size, if the Swin-T model structure remains unchanged, the receptive field cannot expand to cover the entire image before entering the final Swin Transformer block, thus limiting the acquisition of weak semantic prior information. To address this issue, we improved the encoder structure.

**Figure 2:**
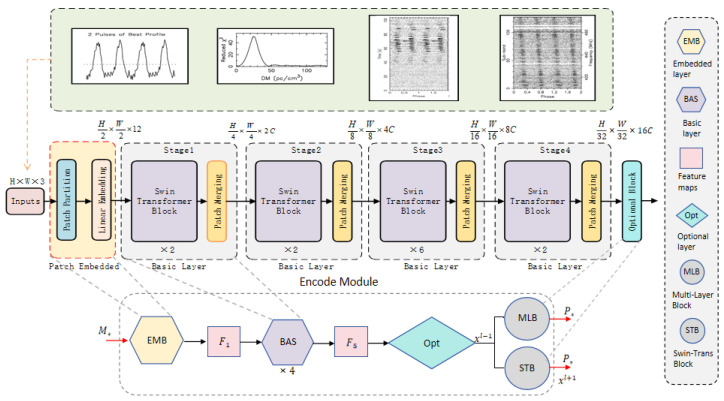
Improvement of encoder structure.

We introduce additional patch merging modules and optional layers in the original encoder (as shown in Figure 2). This modification aims to expand the receptive field of the sliding window, allowing for better capture of global image semantic features. It enhances the feature extraction process while minimizing the increase in computational costs.

The improvements and adjustments to the encoder can be observed from Figure 2, which can be summarized into three steps:

Firstly, before entering the first stage, the patch partition and linear embedding in the standard structure are combined to form a new EMB layer (patch embedding block), preparing for the construction of subsequent basic layers.

Secondly, the positions of subsequent patch merging are shifted backward and combined with Swin Transformer blocks to create BAS layer (basic modules). This approach allows us to add a patch merging module after the first Swin Transformer block, resulting in a window composed of only seven patches after the final patch merging is completed. This method is similar to gradually expanding the receptive field range in a CNN network to cover the entire input signal image.

Additionally, after the last basic layer, an optional module is added, which takes the complete image of the last window as the recognition input. The optional module can have two choices: either adding fully connected an MLB (Multi-Layer Perceptron block) or adding an STB (Swin Transformer block).

The STB block consists of a Window Multi-Head Self-Attention (W-MSA) layer and a Shifted-Window Multi-Head Self-Attention (SW-MSA) layer. The network architecture can be represented with Equations (1)–(4).

(1) $${\hat{x}}^{l}=W-MSA\left(LN\left(x^{l-1}\right)\right)+x^{l-1}$$

(2) $$x^{l}=MLP\left(LN\left({\hat{x}}^{l}\right)\right)+{\hat{x}}^{l}$$

(3) $${\hat{x}}^{l+1}=SW-MSA\left(LN\left(x^{l}\right)\right)+x^{l}$$

(4) $$x^{l+1}=MLP\left(LN\left({\hat{x}}^{l+1}\right)\right)+{\hat{x}}^{l+1}$$

In each basic layer, the feature vector “$x^{l-1}$” is first normalized using layer normalization (LN). It then undergoes feature learning through the Window Multi-Head Self-Attention (W−MSA) layer, followed by a residual operation, resulting in the feature vector “${\hat{x}}^{l}$”. After that, “${\hat{x}}^{l}$” is normalized again using LN, passed through a Multi-Layer Perceptron (MLP), and then undergoes another residual operation. The final output feature vector of the W−MSA layer is denoted as “$x^{l}$”. The structure of the Shifted-Window Multi-Head Self-Attention (SW−MSA) layer is similar to the W−MSA layer. It takes the output feature vector “$x^{l}$” from the W−MSA layer as its input. The input is first normalized using LN, then undergoes feature learning through the SW−MSA layer, followed by a residual operation, resulting in the feature vector “${\hat{x}}^{l+1}$”. After that, “${\hat{x}}^{l+1}$” is normalized again using LN, passed through an MLP, and then undergoes another residual operation. The final output feature vector of this SW−MSA layer is denoted as “$x^{l+1}$”, which serves as the output of the Swin Transformer block.

#### 2.1.2. Cross-Feature Fusion Module

**Figure 3:**
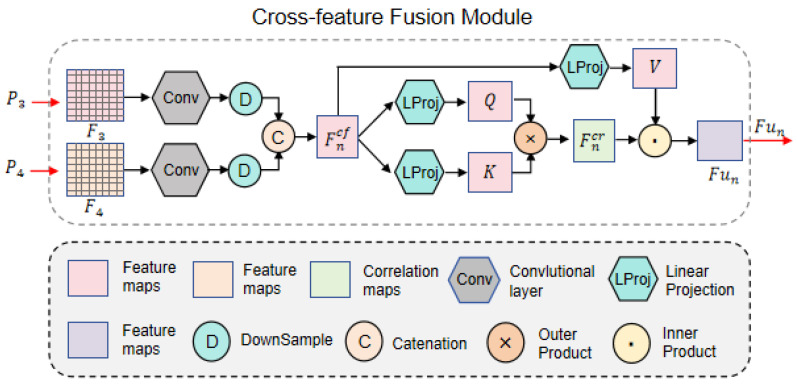
Cross-feature fusion module.

As shown in Figure 1, four categories of candidate subimages serve as inputs. The low-level feature information undergoes patch merging and encoding via the encoder, progressively generating higher-level feature maps $P_{n}\left(n=1,2,3,4\right)$ enriched with semantic information. Notably, the time–phase maps and frequency–phase maps share complementary periodic semantic information. Integrating these two views provides generalized shape-aware details of candidate images, enhancing their feature representation capability and thereby improving the model’s recognition accuracy and generalization performance. Based on this hypothesis, we designed the cross-feature fusion (CFF) module, illustrated in Figure 3.

The CFF module takes the high-level feature maps $F_{3}$ and $F_{4}$, corresponding to the time–phase diagram and frequency–phase diagram, as inputs. Both feature maps have dimensions of 1 × 1 × 768. This matching dimension allows them to be directly fed into the CFF module for fusion. After convolution, downsampling, and concatenation operations, the module derives the cross-feature map $F_{n}^{cf}$:

(5) $$F_{n}^{cf}=\mathrm{Cat}\left[↓\mathrm{Conv}\left(P_{n}\right),\mathrm{Conv}\left(P_{m}\right)\right]$$

where n=3 and m=4, Conv(·) denotes the convolution operation, ↓ represents downsampling, and Cat[·] indicates catenation. Next, to speed up the training process and improve the model’s convergence rate, we performed layer normalization on $F_{n}^{cf}$ and then fed it into the self-attention mechanism module for computation. Through the self-attention mechanism, we calculated the cross-feature prior correlation $F_{n}^{cr}$, which measures the importance of different feature representations and allows the network to focus on the most relevant information for the task at hand. The formula is as follows:

(6) $$\begin{array}{cc} F_{n}^{cr} & =\mathrm{sig}(Q\times K)\\ \; & =\mathrm{sig}\left(\mathrm{Softmax}\left(\frac{LProjQ\left(F_{n}^{cf}\right)\cdot LProjK{\left(\left(F_{n}^{cf}\right)\right)}^{T}}{\sqrt{d_{k}}}\right)\right) \end{array}$$

Here, sig(·) denotes the sigmoid function, “×” represents the outer product operation, and LProjQ(·) and LProjK(·) are linear projections that derive the query vector *Q* and key vector *K*, respectively.

To further enhance feature representations with positional and shape-aware information, the CFF module combines the cross-feature map $F_{n}^{cf}$ and prior correlation $F_{n}^{cr}$ as attention scores. The final output $F_{u}$ of the CFF module is obtained by computing their inner product:

(7) $$\begin{array}{cc} F_{u} & =F_{n}^{cr}\cdot V\\ \; & =F_{n}^{cr}\cdot LProj\left(F_{n}^{cf}\right) \end{array}$$

where “·” denotes the inner product operation, and LProj(·) represents the linear projection that generates the value vector *V*.

In this process, the fused feature map $F_{u}$ is iteratively updated through training on the input features $P_{3}$ and $P_{4}$. $F_{u}$ is then subjected to layer normalization and global average pooling before being fed into the final fully connected layer for decoding. To balance information compression and feature retention while further extracting high-level abstract features, the features pass through two hidden layers with dimensions of 384 and 128, respectively. Finally, softmax is applied at the output layer to generate mutually exclusive prediction probability maps $Y^{*}$, which are then sent to the discriminative output module for further processing.

### 2.2. Discrimination Output Module

In the encode module, the original image *X* is extracted and progressively merged by the encoder to obtain a feature representation map *P* containing 7 × 7 patches of size 32 × 32. After the fully connected layer and the logic layer, probability maps *Y* for each subgraph are generated, and the probability information is then fed into subsequent modules for fusion and discrimination.

The logic layer calculates the weighted probability for each subgraph by multiplying the recognition accuracy of the *i*-th class subimage ($\theta_{i}$) by the weight matrix of the recognition result ($\omega_{ik}$) for that class. Subsequently, the results are normalized. This indicates that subgraphs with higher recognition accuracy have a greater impact on the recognition results:

(8) $$\begin{array}{cc} y_{ik} & =Logit\left(p_{ik}\right)\\ \; & =LN\left(GAP\left(MLP\left(p_{ik}\right)\right)\right)\\ \; & =Norm\left(\theta_{i}\omega_{ik}\right)\\ \; & =\frac{\exp\left(\theta_{i}\omega_{ik}\right)}{\sum_{k=1}^{n}\exp\left(\theta_{i}\omega_{ik}\right)} \end{array}$$

where MLP (Multi-Layer Perceptron) and GAP (Global Average Pooling) represent fully connected feed-forward neural networks and global average pooling operations, respectively, while LN indicates layer normalization. The variable *i* denotes the four different categories of subgraphs, *i* = 1, 2, 3, 4, *k* is the column index of the weight matrix for recognition results, and *k* = 1, 2. The variable $y_{ik}$ represents the weight of the *i*-th class subgraph identified as an interference signal (*k* = 1) and pulsar signal (*k* = 2).

Then, the fusion and discrimination of probability maps are executed through a three-stage process:

A. Fusion of weights

In the first stage, the weights $Y_{2}$ of the subimages $M_{2}$ and the cross-feature fusion weight $Y^{*}$ are fused to obtain the fused weight $Z_{k}$. The weight fusion is performed using the following function:

(9) $$\begin{array}{cc} \; & Z_{k}=Norm\left(y_{2k}+y_{k}^{*}\right) \end{array}$$

where *k* = 1, 2. The value $Z_{k}$ represents the fusion probability of being identified as an interference signal (*k* = 1) and a pulsar signal (*k* = 2).

B. Comparison of weight distance

Next, we employ the concept of Minkowski distance to quantify the difference between the recognition weights of single-class pulsar subimages and the fused weights, thereby establishing correlations among the originally independent result sets of various pulsar candidate subimages.

(10) $$\begin{array}{cc} \; & D\left(y,z\right)={\left(\sum_{j=1}^{N}{\left|y_{j}-z_{j}\right|}^{p}\right)}^{\frac{1}{p}} \end{array}$$

where *p* = 1, 2, *∞*, 1≤j≤N.

In Equation (10), $y_{j}$ and $Z_{j}$ represent the recognition probability map of the *j*-th subimage of a certain class of pulsar candidates and its fused probability weight map, respectively. To facilitate the analysis of the trend in recognition weight changes, it is necessary to quantify this difference. Through experimental comparisons, we found that setting p=2 (i.e., Euclidean distance), the final recognition accuracy is relatively high. This is because a higher *p* value assigns greater weight to the largest differences and less weight to smaller differences, which affects the accuracy of the final category judgment. By calculating the Euclidean distance between the probability weight of the corresponding subgraph and the average fusion weight and comparing them, the similarity coefficients *D* and $D^{*}$ are obtained:

(11) $$\begin{array}{cc} D & =\frac{{∥y_{12}-y_{11}∥}_{2}}{{∥\bar{z_{1}}-\bar{z_{2}}∥}_{2}}\\ \; & =\frac{{∥Norm\left(\theta_{1}\omega_{12}\right)-Norm\left(\theta_{1}\omega_{11}\right)∥}_{2}}{{∥Norm\left(\bar{y_{21}+y_{1}^{*}}\right)-Norm\left(\bar{y_{22}+y_{2}^{*}}\right)∥}_{2}} \end{array}$$

(12) $$\begin{array}{cc} D^{*} & =\frac{{∥y_{11}-y_{12}∥}_{2}}{{∥\bar{z_{2}}-\bar{z_{1}}∥}_{2}}\\ \; & =\frac{{∥Norm\left(\theta_{1}\omega_{11}\right)-Norm\left(\theta_{1}\omega_{12}\right)∥}_{2}}{{∥Norm\left(\bar{y_{22}+y_{2}^{*}}\right)-Norm\left(\bar{y_{21}+y_{1}^{*}}\right)∥}_{2}} \end{array}$$

In the equation, values $y_{11}$ and $y_{12}$ represent the probabilities of the mean pulse profile subimage being classified as RFI (radio frequency interference) and pulsar signals, respectively, while values $Z_{1}$ and $Z_{2}$ denote the corresponding fused probabilities for RFI and pulsar signal identification.

C. Discrimination of results

The result of the weight comparison function reflects the changes in the recognition results of candidate images. By analyzing and determining the similarity coefficients *D* and $D^{*}$, the final result set *I* can be obtained.

The result set *I* is defined as follows:

(13) $$I=(i_{1},i_{2},\ldots ,i_{N})$$

Thus,

∀1≤j≤N

Moreover,

$$If\;\;y_{12}>y_{11}\;\;and\;\;Z_{1}>Z_{2}$$

By comparing the value of *D*, the value of the set *I* is determined:

(14) $$i_{j}=\left\{\begin{array}{ccc} 0, & if & D\leq 1.0\\ b_{1j}, & if & D>1.0 \end{array}\right.$$

Moreover,

$$If\;\;y_{11}>y_{12}\;\;and\;\;Z_{2}>Z_{1}$$

By comparing the value of $D^{*}$, the value of the set *I* is determined:

(15) $$i_{j}=\left\{\begin{array}{ccc} 1, & if & D^{*}\leq 1.0\\ b_{1j}, & if & D^{*}>1.0 \end{array}\right.$$

where $b_{1j}$ denotes the recognition probability of the corresponding subimage in the standard annotated set $B_{1}$.

Specifically, if the recognition weight of a mean pulse profile plot satisfies condition $y_{12}>y_{11}$, it indicates that the subgraph’s recognition result is a pulsar signal (labeled as 1). If the cumulative sum of the recognition weights of the DM curve plot and cross-feature fusion weight satisfies condition $Z_{1}>Z_{2}$, it indicates that a significant portion of the subimages corresponding to these weight components are classified as RFI. In this case, the recognition result of the first subgraph (mean pulse profile) may be incorrect. A comparative analysis of weight distances was conducted using Equations (11) and (12), revealing the variation trends of recognition weights. If we have D≤1.0, this indicates a higher probability that the corresponding subimages represent RFI rather than pulsar signals. In this case, the marking of the mean pulse profile plot will be changed from 1 to 0; otherwise, it remains unchanged. Conversely, if the recognition weight of a certain mean pulse profile plot satisfies condition $y_{11}>y_{12}$, this indicates that the recognition result of that subgraph is an interference signal (labeled as 0). According to similar strategies mentioned above, when we have $D^{*}\leq 1.0$, this indicates a clear tendency that the corresponding subimages represent pulsar signals. Therefore, the label of the corresponding subgraph recognition result will be changed from 0 to 1; otherwise, it remains unchanged. The resulting labeled values will be stored in the final result set *I*.

In Algorithm 1, we present a portion of PyTorch-like pseudocode for implementing our recognition strategy.
**Algorithm 1** CFHAPNet strategy**Input:**  Dataset: $B_{i}$ = ${\left(b_{i1},b_{i2},\ldots ,b_{iN}\right)}^{T}$;   Some constraint information: $b_{ij}$ = 0, 1, *i* = 1, 2, 3, 4, 1≤j≤n;   Parameters: Number of true positive (TP) values and number of false positive (FP) values in $B_{i}$.**Output:**  Datasets *I*. STRATEGY (TP,FP)  Calculate the recall rate R of observation dateset $B_{i}$.  Calculate the weighted probabilities of each subgraph and the fusion weights of the corresponding subgraphs in the order that satisfies condition $R_{B_{1}}\geq R_{B_{2}}\geq R_{B_{3}}\geq R_{B_{4}}$. **for** iinrange1to4 **do**  **for** kinrange1to2 **do**   $y_{ik}\leftarrow Norm\left(\theta_{i}\omega_{ik}\right)$   $Z_{k}\leftarrow Norm\left(y_{2k}+y_{k}^{*}\right)$ **end for****end for**  Calculate the values of *D* and $D^{*}$ via discriminant functions (11) and (12). **if** $\;y_{12}>y_{11}\;and\;Z_{1}>Z_{2}\;$ **then**  *Update the value o f set I Via Equation* (14) **end if** **if** $\;y_{11}>y_{12}\;and\;Z_{2}>Z_{1}\;$ **then**  *Update the value o f set I Via Equation* (15) **end if** **return** *I*

According to the CFHAPNet model structure and recognition strategy, it can be seen that the recognition accuracy of each subgraph has a direct impact on the recognition accuracy of result sets *I*. Next, we aim to further improve the recognition accuracy and stability of the subimages by enhancing the loss function.

### 2.3. Loss Function

In the previous section, we learned that some pulsar plots have low distinguishability from interference signals. By using the center loss function to calculate distances within the same class samples, the feature vectors of the same class samples can cluster around a central point, providing noise resistance while improving the model’s classification accuracy.

**Figure 4:**
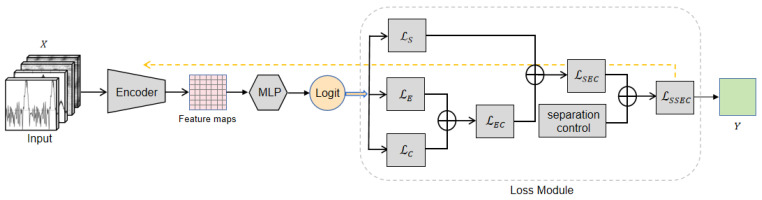
Structure of loss function. By combining the proposed $L_{SS}$ and $L_{EC}$, the introduced $L_{SSEC}$ considers both inter-class distance and intra-class compactness.

However, traditional center loss only considers the Euclidean distance from the center. Although it improves intra-class compactness by minimizing the Euclidean distance between features and their centers, it may not provide sufficient attractiveness for certain samples, especially hard samples. As described by the authors of the center loss in [51], we should not overestimate the capability of center loss. Building upon the Euclidean distance center, the loss function model as shown in Figure 4 is designed to address issues such as insufficient compactness of same-class features and weak separability of features between classes.

#### 2.3.1. Enhancement of Intra-Class Compactness

In Equation (16), we introduce cosine center loss to enhance the attraction of sample centers and utilize a corresponding weighted loss function to supervise training:

(16) $$L=L_{S}+\alpha L_{E}+\beta L_{C}$$

where $L_{E}$ is the center loss, $L_{C}$ is the cosine loss, and beta is its weight. The cosine loss $L_{C}$ is defined as Equation (17):

(17) $$\begin{array}{cc} \; & L_{c}={\left[\frac{1}{m}\sum_{i=1}^{m}C\left(x_{i},c_{y_{i}}\right)\right]}^{\gamma }={\left[1-\frac{1}{m}\sum_{i=1}^{m}C^{\prime }\left(x_{i},c_{y_{i}}\right)\right]}^{\gamma } \end{array}$$

where $x_{i}={\left(x_{i,1},x_{i,2},\ldots ,x_{i,n}\right)}^{T}$ is the feature of the *i*-th image in each batch, $c_{yi}={\left(c_{yi,1},c_{yi,2},\ldots ,c_{yi,n}\right)}^{T}$ is the center of $x_{i}$, and $y_{i}$ is the ground truth of $x_{i}$. Gamma is a positive number greater than 1 used to control the stability of the entire model. $C(x_{i},c_{yi})$ is the cosine distance between $x_{i}$ and $c_{yi}$, and $C^{\prime }\left(x_{i},c_{yi}\right)$ is the cosine similarity between $x_{i}$ and $c_{yi}$, defined as follows:

(18) $$\begin{array}{cc} \; & C^{\prime }\left(x_{i},c_{yi}\right)=\frac{\mathrm{cov}\left(x_{i},c_{y_{i}}\right)}{\sigma x_{i}\cdot \sigma c_{y_{i}}}=\frac{{\left(x_{i}-\bar{x_{i}}\right)}^{T}\left(c_{y_{i}}-\bar{c_{y_{l}}}\right)}{{∥x_{i}-\bar{x_{1}}∥}_{2}{∥c_{y_{i}}-\bar{c_{y_{i}}}∥}_{2}} \end{array}$$

where $\sigma x_{i}$ and $\sigma c_{y_{i}}$ are the standard deviations of $x_{i}$ and $c_{yi}$, respectively. $\bar{x}$ is the mean vector of all components of *x*, with the same size as *x*.

**Figure 5:**
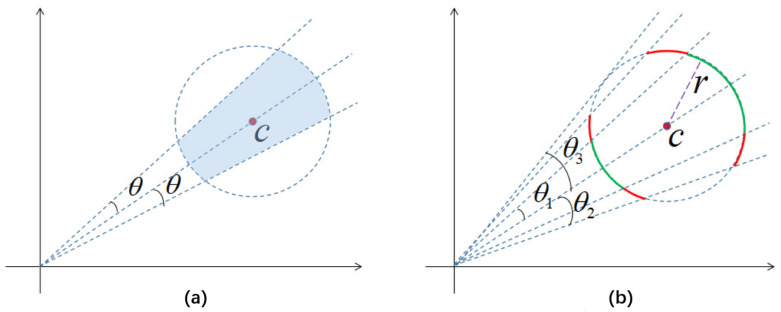
The impact of different γ values on $L_{E}+L_{C}$. (**a**) Effect of The combination of $L_{E}+L_{C}$. (**b**) Effect of different γ.

The reason we incorporate cosine distance into the loss function calculation is mainly due to the strong complementarity between cosine distance and the Euclidean distance primarily used in the original center loss function. Combining $L_{E}$ and $L_{C}$ can confine features of the same class within the intersection of hyperspheres in the feature space (Figure 5a), bringing features closer to their centers and enhancing intra-class cohesion. The value of γ must be greater than 1; otherwise, in the middle to later stages of training, the gradients $\left(\partial L_{c}/\partial x_{i}\right)$ and $\left(\partial L_{c}/\partial c_{i}\right)$ will become very large, causing $L_{SEC}$ to not converge. For the same iteration during the training process (the same radius *r* in Figure 5b), a larger γ leads to a lower correlation between features and centers, resulting in larger angles between features and centers, providing more favorable conditions for training hard samples.

From Figure 5, it can be observed that for a given $1<\gamma_{1}<\gamma_{2}$, when the distance between features and centers is fixed at r (i.e., the same iteration), the features of the model corresponding to $\gamma =\gamma_{1}$ are confined only to the green arc, while the features of the model corresponding to $\gamma =\gamma_{2}$ are jointly confined to the green and red arcs. The angle between features and centers cannot exceed $\theta_{3}$, so the model tends to stabilize when γ exceeds a certain value.

#### 2.3.2. Separation Control

Center loss only considers intra-class compactness of features and does not take into account inter-class separability [52].The inclusion of cross-entropy loss $L_{S}$ is to consider a certain level of inter-class separability during the recognition process. The output of the MLB layer in Figure 2 is essentially the dot product of the fully connected layer parameters (W) and the last layer’s feature vector $x_{i}$, which is $W^{T}x_{i}+b$. Therefore, in order to improve the discriminative ability of the features and increase the gap between them, we remove the traditional cross-entropy loss function $L_{S}$ and introduce a new loss function $L_{SS}$. By applying the margin to the angles and introducing a parameter m to fine-tune the margin, in conjunction with the improvement for the loss function in Section 2.3.1, we train the model using the following loss function:

(19) $$L_{SSEC}=L_{SS}+\alpha L_{E}+\beta L_{C}$$

where

(20) $$\begin{array}{cc} \; & L_{SS}=-\sum_{i=1}^{N}\log\frac{e^{∥w_{yi}∥∥x_{i}∥\varphi \left(\theta_{y_{i}}\right)+b_{y_{i}}}}{e^{∥w_{yi}∥∥x_{i}∥\varphi \left(\theta_{y_{i}}\right)}+\sum_{i!=y_{i}}e^{∥w_{yi}∥∥x_{i}∥\cos\left(\theta_{j}\right)+b_{j}}} \end{array}$$

(21) $$\begin{array}{cc} \; & \varphi \left(\theta \right)={\left(-1\right)}^{k}\;m\cos\left(\lambda \theta \right)-2k \end{array}$$

(22) $$\begin{array}{cc} \; & \theta =\left\lfloor \frac{k\pi }{\lambda },\frac{(k+1)\pi }{\lambda }\right\rfloor \end{array}$$

where θ satisfies $0⩽\theta ⩽\frac{\pi }{\lambda }$, *k* is an integer used to control the interval of the function φ(θ), satisfying 0⩽k⩽λ−1. λ is a positive integer that controls the inter-class margin. In the monotonically decreasing interval of $\cos\left(\lambda \theta \right)$, the larger the λ, the larger the inter-class margin, and the better the model performance. The parameter *m* is used to fine-tune the margin, where m∈R and satisfies $-1⩽m\cos\left(\lambda \theta \right)⩽1$. Due to the periodic variation in the monotonicity of $\cos\left(\lambda \theta \right)$, when λθ continues to increase beyond the required interval range with a constant *k* value, causing a change in the monotonicity of the function, this will lead to a decrease in model performance.

## 3. Experiments

### 3.1. Data Preparation

**Figure 6:**
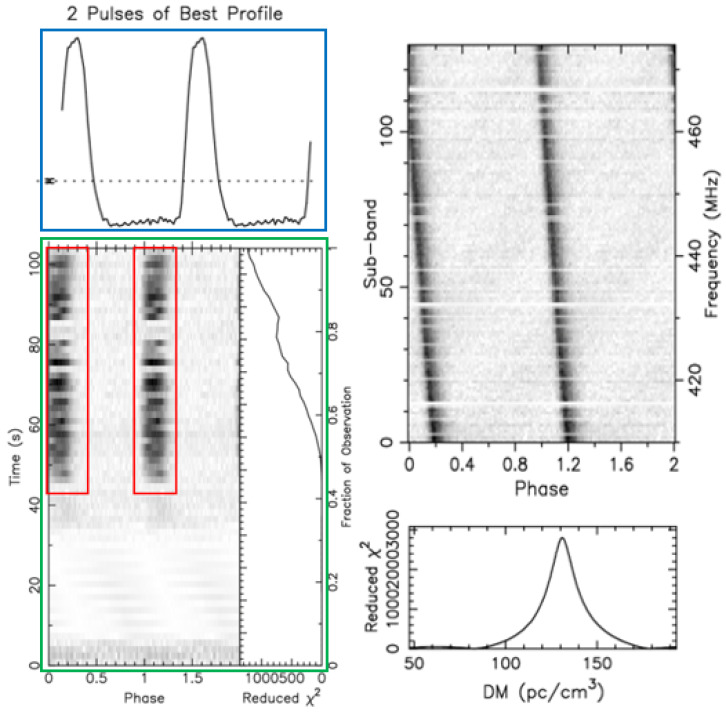
Real pulsar plot obtained from the FAST drift-scan survey.

**Figure 7:**
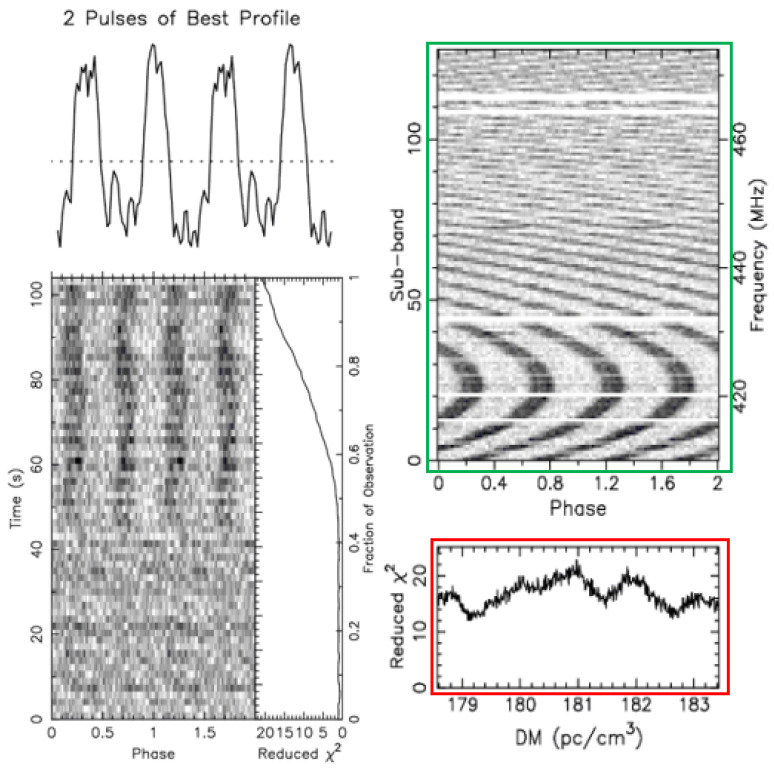
Non-pulsar plot obtained from the FAST data.

We use data obtained from astronomical centers and publicly available datasets for our experiments. The collected signal data were processed using the PulsaR Exploration and Search Toolkit (PRESTO) pipeline software(Version 5.0.0). The resulting candidate diagnostic images have a similar presentation format. The following figures present two examples of a pulsar candidate plot. The positive (pulsar, Figure 6) and negative (non-pulsar, Figure 7) candidates exhibit different characteristics.

Pulsars emit pulses continuously and periodically, but each individual pulse is very weak. By folding the signal at the expected period and stacking consecutive pulses, these faint individual pulses become significantly enhanced and prominent, showing up as a bright line at the same phases (red box in Figure 6). This bright line should also match the periodic pattern of the pulse curve in the average pulse profile plot (blue box in Figure 6). Figure 6 is a time–phase relationship plot (green box) of a real pulsar, showing periodic intermittent signals caused by pulsar beam drifting.

Pulsar radiation pulses consist of multiple radio frequencies. As these radio waves travel through the interstellar medium, different frequencies arrive at Earth at slightly different times due to dispersion. We measure this effect using the Dispersion Measure (DM). For a genuine pulsar, a specific DM range should correspond to a single strong signal.

In Figure 7, the frequency–phase plot (green box) shows periodic features similar to those of pulsar signals. However, the DM curve (red box) does not have the smooth single-peak shape that most pulsar DM curves have, as seen in Figure 6. This means many DM values correspond to multiple peak signals, indicating radio signals coming from different directions and distances. This suggests the signals are likely from terrestrial interference, and the candidate is not a pulsar.

The processed candidate image dataset is annotated and divided it into a training and testing set to train the CFHAPNet model. In addition to FAST data, the HTRU dataset originates from observations conducted by the Parkes Telescope in Australia using multiple beams (13 beams). Table 1 displays the number of positive and negative examples in each dataset.

### 3.2. Training Configurations

We use the improved Swin Transformer as the backbone network and pretrain the model on the FAST dataset. During both training and testing phases, the input images are processed as follows: (1) resize the image to 224 × 224; (2) linearly adjust pixel values to [0, 1]; and (3) set mean and standard deviation to [0.485, 0.456, 0.406] and [0.229, 0.224, 0.225], respectively. We employ the Adam parameter optimization strategy, and we set the batch size to 8, the number of epochs to 50, and the learning rate to 0.0001. Similar to [53], we set α to 0.003. The experimental hardware environment consists of a Tesla V100 GPU, Video Memory 16 GB, and RAM 32 GB. The software environment includes Windows 10, CUDA Toolkit 11.7, Python 3.9.12, and PyTorch 1.11.0.

### 3.3. Evaluation Metrics

Precision, recall, and F1-score are used for the quantitative evaluation of each model’s performance. mAP and CMC1 are primarily used to assess the impact of the loss function on model performance. Assuming that the precision and recall of the subgraph sgi among the top k images identified from a certain class of subimages are P(${\mathrm{sg}}_{i}$,k) and R(${\mathrm{sg}}_{i}$,k), respectively, and that P(${\mathrm{sg}}_{i}$,0) = 0 and R(${\mathrm{sg}}_{i}$,0) = 0, the average precision (AP) and mean average precision (mAP) are defined as [54,55,56]:

(23) $$\begin{array}{cc} \; & \mathrm{AP}\left({\mathrm{sg}}_{i}\right)=\sum_{k=1}^{G}P\left({\mathrm{sg}}_{i},k\right)\delta \left[P\left({\mathrm{sg}}_{i},k\right)\neq P\left({\mathrm{sg}}_{i},k-1\right)\right] \end{array}$$

(24) $$\begin{array}{cc} \; & \mathrm{mAP}=\frac{\sum_{i=1}^{Q}\mathrm{AP}\left({\mathrm{sg}}_{i}\right)}{Q} \end{array}$$

where Q and G represent the total number of images in the query set and the subimage set, respectively, and for δ(·), there are δ(True) = 1 and δ(False) = 0.

Cumulative Matching Characteristics (CMC@k) [51,57] can comprehensively reflect the performance of the classifier. It represents the ratio of the number of correctly identified labels among the top *k* items to the total number of test samples, defined as follows:

(25) $$\begin{array}{cc} \; & \mathrm{CMC}@k=\frac{\sum_{i=1}^{Q}h\left({\mathrm{sg}}_{j}k\right)}{Q} \end{array}$$

where Q is the total number of images in the query set, and $sg_{i}$ is the *i*-th image in the query set. If there is a correct query $sg_{i}$ among the top *k* items, then h($sg_{i}$, k) = 1; otherwise, h($sg_{i}$, k) = 0.

## 4. Results

### 4.1. Comparison with SOTA Methods

In practical applications, the CFHAPNet model and filtering strategy were initially used for the FAST dataset. Subsequently, transfer learning was applied to fine-tune the parameters of the model on PMPS and HTRU data, ensuring that the model can better adapt to more application scenarios. By comparing CFHAPNet with previous research methods, we aim to understand its advantages and disadvantages and validate its generalization performance. The experimental results are shown in Table 2.

**Figure 8:**
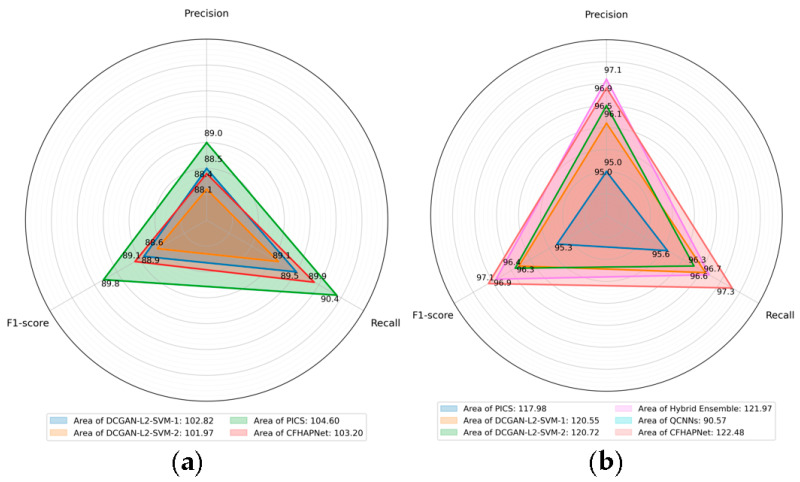
Performance comparison of different methods. (**a**) Comparison on the PMPS dataset. (**b**) Comparison on the HTRU dataset.

To comprehensively compare different methods (using the same dataset as our experiments), Figure 8 shows the radar charts of three important metrics obtained by each method to emphasize the differences in method performance. Additionally, the numbers indicated in the legend represent the area of the region enclosed by the three metrics; the larger the area, the better the performance of the method.

### 4.2. Ablation Results

#### 4.2.1. CFHAPNet Model Validation

Firstly, to understand the impact of the features of each subimage on the recognition results, experiments are conducted to recognize each subimage without using the CFHAPNet model. Due to the significant differences in the features of each subimage, it is found that the overall accuracy, recall rate, and precision rate of each subimage recognition all vary (Table 3). The mean pulse profile and DM curve have a higher recall rate but lower precision rate and overall accuracy. This indicates that these two feature images can identify most of the pulse signals but also identify many interference signals as pulsars. As is seen from Figure 6 and Figure 7, for the time–phase plot and frequency–phase plot of pulsars, there are often regular vertical lines running through the feature images; these two feature images of non-pulsars often do not have vertical lines running through or the signals show other forms such as curves. This ensures that these two features can ensure a certain overall recognition rate and precision, but the recall rate is significantly reduced.

Subsequently, without encoder optimization, we compared the performance of the CFHAPNet model’s hybrid connection strategy against simple multi-subimage mosaicking recognition using the FAST dataset, with experimental results summarized in Table 4. The results demonstrate that employing CFHAPNet for hybrid connections improves precision, recall, F1-score, and Top-1 accuracy by 3.32%, 1.54%, 2.45%, and 1.81%, respectively. This validates the effectiveness of CFHAPNet’s hybrid connection strategy in enhancing recognition accuracy.

Additionally, when experiments were conducted by connecting four types of subgraphs in reverse order, we observed a significant degradation in all evaluation metrics compared to the results obtained using the original connection order adopted by the CFHAPNet model. This demonstrates the validity of our network architecture design and recognition strategy.

#### 4.2.2. Structural Improvements of Encoder

To improve the recognition accuracy of each subimage, the role of each component in the encoder is investigated. Since the encoder is based on the Swin Transformer architecture, we conducted ablation experiments by progressively redesigning components starting from the base model to demonstrate their importance in enhancing performance. We focus on issues such as patch merging and downsampling operations, hoping to improve accuracy while minimizing the increase in processing time as much as possible.

Firstly, the downsampling rate is adjusted to H/2, W/2. After each downsampling, we can obtain 12,544 feature vectors of length 12. This ensures that the detailed information of the signal image is captured as much as possible without changing the total length of the feature vectors. As shown in Table 5, the recognition accuracy of each subimage improved to varying degrees compared to the base model with a downsampling rate set to H/4 and W/4. In particular, the recognition accuracy of the result set I increased by 0.8%, reaching 93.82%.

As can be seen from Table 5, when the optional layers are implemented as STB and MLB, the recognition accuracy for Result Set *I* reaches 94.41% and 95.38%, respectively. With the integration of the CFF module, the accuracy is further improved to 97.23%, representing a 4.15% enhancement compared to the baseline architecture.

**Figure 9:**
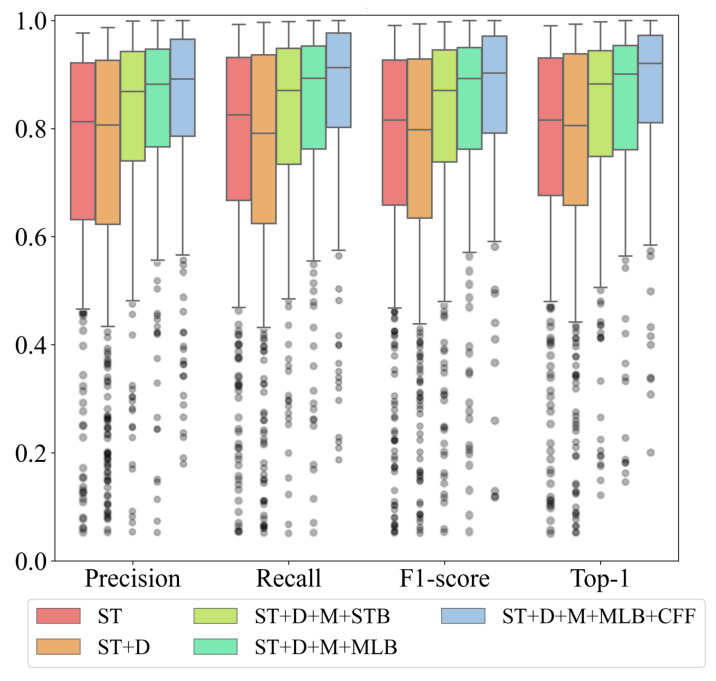
Box plots of different evaluations metrics for ablation results on the test set (FAST). Box plots of different evaluations metrics for ablation results on the test set (FAST). ST+D+M+MLB+CFF is the model with the patch merging, optional MLB layer and cross-feature fusion module. ST+D+M+MLB is the model with the patch merging and optional MLB layer. ST+D+M+STB is the model with the patch merging and optional STB layer. ST+D is the basic Swin-T model using the new downsampling scheme but does not include any optional layers. ST is the basic Swin-T model.

To quantitatively show the impact of various improvement measures, Figure 9 presents box plots of the main evaluation metrics obtained from different ablation experiments. We find that after adopting the new downsampling strategy, although the upper quartiles (Q1) of all metrics slightly improved by 1.35% to 2.45%, the lower quartile (Q3) positions of the corresponding metrics also decreased by 0.82% to 4.28%. This indicates that merely adjusting the downsampling rate to capture more image detail is indeed beneficial for improving recognition metrics. However, due to the limitations imposed by the final image receptive field, the fluctuation in indicator data increases, leading to unstable recognition performance. The introduction of an additional optional layer and CFF feature fusion module significantly reduces the range of metric fluctuations, thereby markedly improving recognition stability. The median and third quartile of each metric show considerable enhancement compared to the basic Swin-T model. Notably, regarding the Top-1 metric, when introducing the CFF module while adopting MLB as the optional layer, the improvement becomes particularly pronounced compared to the baseline Swin-T. Beyond an approximately 4% increase in the first quartile (Q1), the second and third quartiles (Q2 and Q3) exhibit enhancements of 10.53% and 13.49%, respectively. This indicates that these improvement measures enable the model to achieve optimal performance across the majority of samples.

### 4.3. Improvements of Loss Functions

#### 4.3.1. Ablation Results

In Section 2.3, we discussed two drawbacks of the original loss: (1) it does not consider inter-class separability and (2) the attraction of hard samples is insufficient. In fact, the solutions to these two drawbacks are essentially the same because as long as the inter-class distance is increased sufficiently, thus considering inter-class separability, the sample points will also be more concentrated around the sample centers of their respective classes. Therefore, the proposed Lssec simultaneously addresses these two drawbacks.

We compared the results of $L_{SSEC}$ with the loss functions $L_{E}$ and $L_{S}$ on the FAST dataset. Simultaneously, we also removed $L_{C}$ from $L_{SSEC}$ (using $L_{SS}$+$L_{E}$) in the experiment to verify the effectiveness of $L_{C}$. To demonstrate the generalization ability of $L_{SSEC}$, we conducted the above experiments on other two major datasets in the pulsar recognition field, PMPS and HTRU. The experimental results are shown in Table 6, Table 7 and Table 8.

From Table 6, Table 7 and Table 8, it can be seen that compared to $L_{E}$, in these three datasets, all experimental results using only $L_{E}$ for supervising training are poor. Since the model’s parameters are randomly assigned by the system without any training, this indicates that $L_{E}$ cannot be used for supervising training without $L_{S}$. On the other hand, all experimental results of our proposed $L_{SSEC}$ have reached high levels, far exceeding those using only $L_{E}$. This indicates that our $L_{SSEC}$ can indeed train the model independently.

Compared to $L_{SSEC}$, softmax loss ($L_{S}$) does not consider intra-class compactness or inter-class separability; it only focuses on the overall classification ability of the model. From Table 6, Table 7 and Table 8, we can observe that $L_{SSEC}$’s MAP and CMC1s in all three datasets far exceed those of $L_{S}$. These results indicate that considering intra-class and inter-class distances, $L_{SSEC}$ is indeed very helpful in improving recognition performance.

The experimental results also indicate that when we remove $L_{C}$ from $L_{SSEC}$ ($L_{SSE}$), focusing solely on enhancing inter-class separability can effectively allow us to supervise the training. However, from Table 6, Table 7 and Table 8, it can be seen that in all subfigures of the datasets, the mAP of $L_{SSEC}$ is approximately 10–13% higher than that of $L_{SSE}$, and CMC1 is about 8–12% higher than $L_{SSE}$. This indicates that the proposed $L_{C}$ is highly effective.

**Figure 10:**
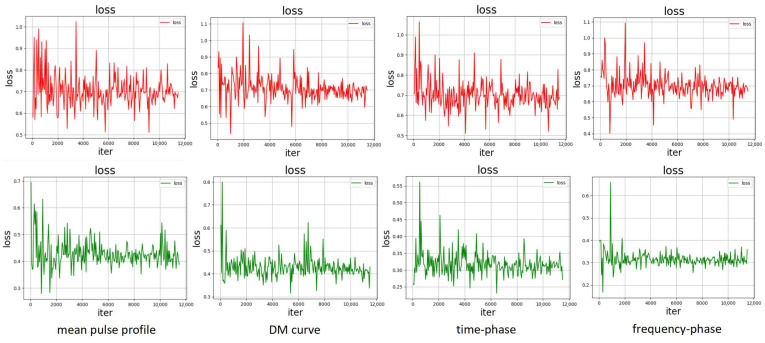
Comparison of loss function convergence. The graphs are obtained with the parameter settings of epoch number = 50 and learning rate = 0.0001.

Furthermore, we visualized the convergence behavior when performing recognition of the four types of subimages using $L_{SSEC}$ (in green) compared to the original loss function $L_{SE}$ (in red) in Figure 10.

From the experimental results, it is evident that the curves of images using $L_{SSEC}$ converge much faster than when using the original loss function. This indicates that the convergence of loss functions for each subfigure significantly improves when using $L_{SSEC}$, thereby enhancing the accuracy and stability of recognition. We also observed that due to the higher similarity in the mean pulse profile images of pulsar signals and interference signals, the improvement in convergence is not as pronounced as in the other three subfigures. This aligns with the data features in the corresponding subfigures in Table 3. Generally, images with better convergence often exhibit higher recognition accuracy. As a result, the convergence speed is accelerated, while classification accuracy and model stability are significantly improved.

Meanwhile, the loss function is applied to the CFHAPNet. We compared the improvement in recognition accuracy of various subgraphs of pulsars using the original loss function and our proposed loss function. As shown in Table 9, the recognition accuracy of each subgraph improved by around 0.8% to 2%. The overall recognition accuracy of the final result set *I* also showed a significant improvement.

#### 4.3.2. Generalization Results

Finally, experiments are conducted on various datasets listed in Table 10. It can be observed that despite differences in preset signal acquisition parameters, sample sizes, and image resolution across datasets, resulting in variations in experimental outcomes, the recognition accuracy rate remains above 89% for all datasets. Notably, on datasets with higher data quality, such as HTRU and FAST, the accuracy even exceeds 97%. These results demonstrate that, under the influence of the proposed loss function, the model exhibits strong stability and generalization performance.

## 5. Discussion

To enhance the recognition efficiency of pulsar candidate images while ensuring good generalization performance, we propose a CFHAPNet model to identify four types of subimages of pulsar candidates and establish logical associations through corresponding strategies. Specifically, we first use a Swin Transformer-based encoder to recognize each subimage and track the feature weight mappings of the subimages. Subsequently, after two stages of feature fusion and comparison of feature weight distances, we discriminate the categories of the candidates. During this process, we optimize the encoder and the loss function to improve the recognition accuracy of the candidate images. Finally, through comparisons with SOTA methods from different signal source datasets and ablation experiments, we validate that the proposed CFHAPNet model and the feature weight tracking and fusion scheme can be effectively used for the recognition of pulsar candidates.

Notably, our study represents the first attempt to achieve associative recognition using heterogeneous multi-channel subimages. Unlike the FAST dataset, each image in the HTRU dataset comprises three distinct channels: Channel 1 represents the period-dispersed subimage, Channel 2 corresponds to the frequency–phase subimage, and Channel 3 represents the time–phase subimage. When processing subimages from these datasets, we exclusively utilized the CFF module within the CFHAPNet encoder for cross-channel feature integration, eliminating the need for additional feature fusion at the discriminative output stage.

As demonstrated in Table 2 and Figure 8, the CFHAPNet model achieves upper-mid to high performance across all metrics: precision (97.5%), recall (98.4%), and F1-score (98.0%). Remarkably, these results surpass recognition levels attained by generative networks trained with extensive positive sample augmentation.

In the ablation experiment section, from Table 5 and Figure 9, we can observe that when the optional layer is MLB, there is a greater improvement in various recognition metrics compared to STB. This is primarily because the number of positive samples is relatively small, and STB is more suitable for processing large-scale data. As the number of samples increases, the recognition accuracy of STB will significantly improve. However, we can also see that when using STB as the optional layer, the processing time for each batch of data is shorter. This means that when the dataset is larger, STB will have higher execution efficiency. Researchers can flexibly adjust the optional layers based on the scale of different datasets.

Additionally, it is important to note that by adopting subgraph recognition and utilizing the proposed model and recognition strategy, we have established logical correlations among the previously independent recognition results. This opens up possibilities for parallel processing of various pulsar subgraphs in the future. From the experimental results in Table 5, we can anticipate that this will significantly reduce the processing time for recognizing massive pulsar data and improve the efficiency of pulsar recognition. This is also one of the original motivations for our research on pulsar subgraph recognition.

From Table 6, Table 7 and Table 8 and Figure 10, it can be observed that our improvements to the loss function take into account the intra-class distances and inter-class separability of the sample features. Apart from the β weight of $L_{C}$ and the λ parameter in $L_{SS}$, no additional parameters were introduced, ensuring computational efficiency. The introduction of the λ parameter expands the inter-class distance, resulting in faster convergence speed and more accurate sample classification.

**Figure 11:**
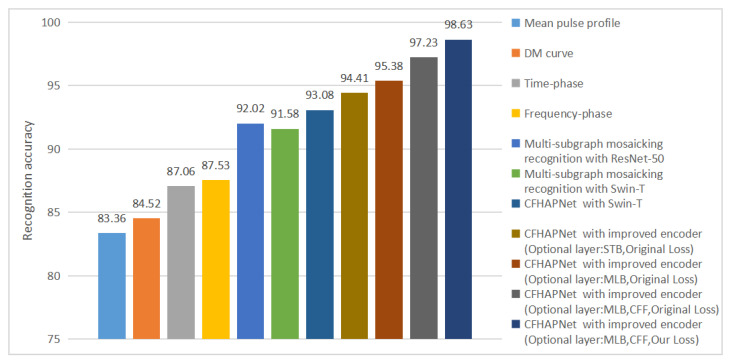
Trend of changes in recognition accuracy.

**Figure 12:**
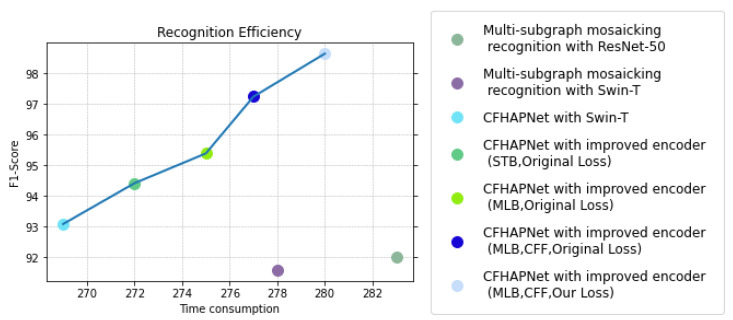
Efficiency comparison of different recognition schemes.

To further analyze and discuss the improvements in recognition performance brought by the CFHAPNet and related enhancements, we visualize and compare the recognition performance of various network architectures applied to single subimage recognition and multi-subgraph mosaicking recognition. Figure 11 reflects the trend of recognition accuracy changes, while Figure 12 compares the recognition efficiency of different network recognition schemes.

As evident from Figure 11, the recognition accuracy for Result Set *I* using CFHAPNet significantly outperforms that of multi-subgraph mosaicking recognition. The accuracy is progressively enhanced with the incorporation of optional layers and the CFF module. Notably, when adopting MLB as the optional layer, the accuracy surpasses that of the standard Swin-T encoder by approximately 4.2%. Furthermore, the proposed loss function in this work improves the recognition accuracy by an additional 1.4%.

As shown in Figure 12, under the same hardware environment, the CFHAPNet model achieves lower per-step processing times compared to multi-subimage mosaicking recognition schemes using ResNet-50 and Swin-T across most encoder optimization configurations; while the inclusion of the CFF module slightly increases processing time, the slope of the performance curve steepens significantly, indicating that the recognition scheme with the CFF module attains a higher time/accuracy efficiency ratio.

Figure 11 and Figure 12 further reveal that when implementing encoder modifications, the recognition time increases slightly by 3 ms (for the optional layer: STB) and 6 ms (for the optional layer: MLB) compared to the standard Swin-T-based recognition scheme; while the recognition accuracy with STB is marginally lower than that of MLB, both configurations achieve nearly identical time-accuracy efficiency ratios.

In summary, compared to CFHAPNet using the standard Swin-T architecture, the improvements made to the encoder and the loss function allow us to achieve a significant increase in recognition accuracy (approximately 5.6%) with a relatively small time cost. This indicates that these enhancements have enabled the CFHAPNet model to achieve a good balance between recognition accuracy and time efficiency.

## 6. Conclusions

A cross-feature hybrid associative priori network for pulsar candidate screening and recognition based on multi-view information is proposed. This model consists of three interconnected modules: an encoding module designed to extract fine-grained patch-level features from raw input images, a cross-feature fusion module that dynamically tracks feature mappings of subimages and performs relevant feature fusion during recognition, and a discriminative output module that combines prior weight mappings and calculates weight distances to generate reliable classification decisions. Comparative evaluations and ablation studies under similar data scales demonstrate that the proposed framework outperforms existing state-of-the-art approaches. Furthermore, the refined loss function enhances model stability and generalization by effectively balancing feature separability and intra-class cohesion. In the long run, these improvements provide a powerful and flexible solution. They allow our research method to keep up with the development of sensor technology and keep providing strong support for pulsar screening. This also helps expand the application potential of our method in large-scale radio astronomical surveys.

## Figures and Tables

**Table 1:** Number of positive and negative examples in the datasets.

Dataset Names	Positive	Negative	Total
Train			
PMPS	700	7000	7700
HTRU	900	8500	9400
FAST	637	9658	10,295
Test			
PMPS	200	3000	3200
HTRU	296	2000	2296
FAST	123	998	1121

**Table 2:** Evaluation of different methods.

Methods	Datasets	Precision	Recall	F1-Score	Positive	Negative
DCGAN-L2-SVM-1 (Guo et al., 2019 [27])	PMPS	88.5	89.5	88.9	1500 RP + 8500 GP	10,000
DCGAN-L2-SVM-2 (Guo et al., 2019 [27])	PMPS	88.1	89.1	88.6	1500 RP + 8500 GP	10,000
PICS (Zhu et al., 2014 [21])	PMPS	89.0	90.4	89.8	2000	10,000
CFHAPNet	PMPS	88.4	89.9	89.1	900	10,000
PICS (Zhu et al., 2014 [21])	HTRU	95.0	95.6	95.3	1196	10,500
DCGAN-L2-SVM-1 (Guo et al., 2019 [27])	HTRU	96.1	96.6	96.3	696 RP + 9304 GP	10,000
DCGAN-L2-SVM-2 (Guo et al., 2019 [27])	HTRU	96.5	96.3	96.4	696 RP + 9304 GP	10,000
Hybrid Ensemblet (Y. Wang et al., 2019 [26])	HTRU	97.1	96.7	96.9	1196	10,500
QCNNs (Donovan Slabbert et al., 2023 [29])	HTRU2	95.0	73.4	82.8	1639	14,620
CFHAPNet	HTRU	96.9	97.3	97.1	1196	10,500

**Table 3:** Recognition results of each subgraph using the standard Swin-T architecture.

Subplot Categories	Image Size	Precision	Recall	F1-Score	Top-1	Ms/Step
Mean pulse profile	224^2^	81.38	88.82	84.94	83.36	66
DM curve	224^2^	82.14	87.72	84.84	84.52	64
Time–phase	224^2^	86.87	86.74	86.79	87.06	70
Frequency–phase	224^2^	87.58	86.38	86.98	87.53	69

**Table 4:** Identification results using CFHAPNet model.

Result Set	Image Size	Precision	Recall	F1-Score	Top-1
Mosaicking	224^2^	88.84	91.61	90.20	91.27
Hybrid connection	224^2^	92.16	93.15	92.65	93.08
Reverse	224^2^	88.42	88.77	88.59	89.61

Identification results using CFHAPNet model. “Mosaicking” refers to a widely used technique in astronomy for stitching multiple non-overlapping images together to form one large composite image, and the “Reverse” row indicates the recognition data obtained by reversing the network structure. This means that the network performs step-by-step filtering of the signal images, starting from the lowest recall rate and highest precision rate according to the subgraph.

**Table 5:** Improved recognition results.

Backbone	Image Size	Precision	Recall	F1-Score	Top-1	Ms/Step
Mean pulse profile plot						
Swin-T Our Downsampler +1 patch merging & STB +1 patch merging & MLB	224^2^ 224^2^ 224^2^ 224^2^	81.38 81.41 81.52 81.92	88.82 88.90 89.06 89.35	84.94 85.00 85.12 85.47	83.36 83.65 83.94 84.24	66 66 66 68
DM curve plot						
Swin-T Our Downsampler +1 patch merging & STB +1 patch merging & MLB	224^2^ 224^2^ 224^2^ 224^2^	82.14 82.32 82.94 83.45	87.72 87.70 87.91 87.95	84.84 84.92 85.35 85.64	84.52 84.88 85.17 85.37	64 65 65 66
Time–phase plot						
Swin-T Our Downsampler +1 patch merging & STB +1 patch merging & MLB	224^2^ 224^2^ 224^2^ 224^2^	86.87 87.02 87.40 87.98	86.72 86.80 87.05 87.21	86.79 86.91 87.23 87.59	87.06 87.69 88.26 89.18	70 68 70 72
Frequency–phase plot						
Swin-T Our Downsampler +1 patch merging & STB +1 patch merging & MLB	224^2^ 224^2^ 224^2^ 224^2^	87.58 87.75 88.12 88.73	86.38 86.40 86.51 86.83	86.98 87.07 87.31 87.77	87.53 88.02 88.65 89.58	69 69 70 70
Result Set *I*						
Swin-T Our Downsampler +1 patch merging & STB +1 patch merging & MLB +CFF Module	224^2^ 224^2^ 224^2^ 224^2^ 224^2^	92.16 92.62 94.25 94.71 96.54	93.15 93.61 94.84 95.25 97.67	92.65 92.86 94.54 94.98 97.10	93.08 93.82 94.41 95.38 97.23	269 270 272 275 277

**Table 6:** Comparing $L_{SSEC}$, center loss, and softmax on the FAST dataset.

Method	Mean Pulse Profile		DM Curve		Time–Phase		Frequency–Phase	
	mAP	CMC1	mAP	CMC1	mAP	CMC1	mAP	CMC1
$L_{E}$	0.244	0.275	0.268	0.288	0.342	0.356	0.274	0.299
$L_{S}$	0.513	0.541	0.546	0.559	0.583	0.605	0.587	0.614
$L_{SSE}$	0.712	0.747	0.724	0.755	0.731	0.776	0.741	0.798
$L_{SSEC}$	0.816	0.834	0.818	0.849	0.855	0.882	0.867	0.894

**Table 7:** Comparing $L_{SSEC}$, center loss, and softmax on the PMPS dataset.

Method	Mean Pulse Profile		DM Curve		Time–Phase		Frequency–Phase	
	mAP	CMC1	mAP	CMC1	mAP	CMC1	mAP	CMC1
$L_{E}$	0.158	0.166	0.166	0.181	0.195	0.205	0.191	0.206
$L_{S}$	0.421	0.457	0.455	0.470	0.476	0.491	0.487	0.508
$L_{SSE}$	0.618	0.644	0.622	0.657	0.644	0.659	0.653	0.677
$L_{SSEC}$	0.691	0.727	0.726	0.738	0.766	0.784	0.784	0.799

**Table 8:** Comparing $L_{SSEC}$, center loss, and softmax on the HTRU dataset.

Method	Mean Pulse Profile		DM Curve		Time–Phase		Frequency–Phase	
	mAP	CMC1	mAP	CMC1	mAP	CMC1	mAP	CMC1
$L_{E}$	0.214	0.246	0.236	0.268	0.275	0.296	0.251	0.276
$L_{S}$	0.508	0.527	0.515	0.531	0.556	0.571	0.557	0.578
$L_{SSE}$	0.708	0.734	0.717	0.736	0.726	0.748	0.734	0.752
$L_{SSEC}$	0.771	0.802	0.785	0.816	0.835	0.857	0.837	0.869

**Table 9:** The impact of different loss functions on recognition results.

Loss Function	Mean Pulse Profile	DM Curve	Time–Phase	Frequency–Phase	Result Set *I*
Original loss	84.24	85.37	89.18	89.58	97.23
$L_{SSEC}$	84.98	86.02	90.84	91.52	98.63

**Table 10:** Recognition results of each dataset.

Datasets	Image Size	Precision	Recall	F1-Score	Top-1
PMPS	224^2^	88.40	89.91	89.15	89.52
HTRU	224^2^	96.91	97.30	97.10	97.40
FAST	224^2^	97.52	98.43	97.97	98.63

## Data Availability

The publicly available FAST data can be obtained at https://github.com/dzuwhf/FAST_label_data (accessed on 25 June 2025), the official FAST data center website and the HEASARC archive website of NASA GSFC.

## Abbreviations

The following abbreviations are used in this manuscript:

CFHAPNet	Cross-feature hybrid associative priori network
FAST	Five-hundred-meter Aperture Spherical Telescope
ConvNets	Convolutional neural networks
ViT	Vision Transformer
Swin-T	Swin Transformer
SOTA	State of the art
CFF	Cross-feature fusion
mAP	Mean average precision
CMC	Cumulative Matching Characteristics

## References

[1] Thanu S., Subha V. (2023). Emerging Trends In Pulsar Star Studies: A Synthesis Of Machine Learning Techniques In Pulsar Star Research. Ann. Comput. Sci. Inf. Syst..

[2] Sureja S., Gadhia B. (2023). Advances in Pulsar Candidate Selection: A Neural Network Perspective. J. Soft Comput. Paradig..

[3] Zhang H., Wang J., Zhang Y., Du X., Wu H., Zhang T. (2023). Review of artificial intelligence applications in astronomical data processing. Astron. Tech. Instruments.

[4] Hobbs G.B., Bailes M., Bhat N.D.R., Burke-Spolaor S., Champion D.J., Coles W., Hotan A., You X.P. (2009). Gravitational-wave detection using pulsars:status of the parkes pulsar timing array project. Publ. Astron. Soc. Aust..

[5] Sengar R., Bailes M., Balakrishnan V., Bernadich M.I., Burgay M., Barr E.D., Wongphechauxsorn J. (2023). Discovery of 37 new pulsars through GPU-accelerated reprocessing of archival data of the Parkes multibeam pulsar survey. Mon. Not. R. Astron. Soc..

[6] Wongphechauxsorn J., Champion D.J., Bailes M., Balakrishnan V., Barr E.D., Bernadich M.I., van Straten W. (2024). The High Time Resolution Universe Pulsar survey–XVIII. The reprocessing of the HTRU-S Low Lat survey around the Galactic Centre using a Fast Folding Algorithm pipeline for accelerated pulsars. Mon. Not. R. Astron. Soc..

[7] Parent E., Kaspi V.M., Ransom S.M., Freire P.C.C., Brazier A., Camilo F., Zhu W.W. (2019). Eight millisecond pulsars discovered in the arecibo PALFA survey. Astrophys. J..

[8] Lynch R.S., Boyles J., Ransom S.M., Stairs I.H., Lorimer D.R., McLaughlin M.A., Van Leeuwen J. (2013). The Green Bank Telescope 350 MHz Drift-scan Survey II: Data Analysis and the Timing of 10 New Pulsars, Including a Relativistic Binary. Astrophys. J..

[9] Swiggum J.K., Pleunis Z., Parent E., Kaplan D.L., McLaughlin M.A., Stairs I.H., Surnis M. (2023). The Green Bank North Celestial Cap Survey. VII. 12 New Pulsar Timing Solutions. Astrophys. J..

[10] Van Der Wateren E., Bassa C.G., Cooper S., Grießmeier J.M., Stappers B.W., Hessels J.W.T., Wucknitz O. (2023). The LOFAR Tied-Array All-Sky Survey: Timing of 35 radio pulsars and an overview of the properties of the LOFAR pulsar discoveries. Astron. Astrophys..

[11] Zhang B., Shang W., Gao X., Li Z., Wang X., Ma Y., Li Q. (2024). Synthetic design and analysis of the new feed cabin mechanism in Five-hundred-meter Aperture Spherical radio Telescope (FAST). Mech. Mach. Theory.

[12] Smits R., Kramer M., Stappers B., Lorimer D.R., Cordes J., Faulkner A. (2009). Pulsar searches and timing with the square kilometre array. Astron. Astrophys..

[13] Labate M.G., Waterson M., Alachkar B., Hendre A., Lewis P., Bartolini M., Dewdney P. (2022). Highlights of the square kilometre array low frequency (SKA-LOW) telescope. J. Astron. Telesc. Instruments Syst..

[14] Ransom S. Presto [EB/OL]. https://www.cv.nrao.edu/~sransom/presto/.

[15] Cai N., Han J.L., Jing W.C., Zhang Z., Zhou D., Chen X. (2023). Pulsar Candidate Classification Using a Computer Vision Method from a Combination of Convolution and Attention. Res. Astron. Astrophys..

[16] Sett S., Bhat N.D.R., Sokolowski M., Lenc E. (2023). Image-based searches for pulsar candidates using MWA VCS data. Publ. Astron. Soc. Aust..

[17] Salal J., Tendulkar S.P., Marthi V.R. (2024). Identifying pulsar candidates in interferometric radio images using scintillation. Astrophys. J..

[18] You Z.Y., Pan Y.R., Ma Z., Zhang L., Zhang D.D., Li S.Y. (2024). Applying hybrid clustering in pulsar candidate sifting with multi-modality for FAST survey. Res. Astron. Astrophys..

[19] Wang Y., Zheng J., Pan Z., Mingtao L.I. (2018). An Overview of Pulsar Candidate Classification Methods. J. Deep. Space Explor..

[20] Tariq I., Qiao M., Wei L., Yao S., Zhou C., Ali Z., Spanakis-Misirlis A. (2022). Classification of pulsar signals using ensemble gradient boosting algorithms based on asymmetric under-sampling method. J. Instrum..

[21] Zhu W.W., Berndsen A., Madsen E.C., Tan M., Stairs I.H., Brazier A., Venkataraman A. (2014). Searching for pulsars using image pattern recognition. Astrophys. J..

[22] Lyon R.J., Stappers B.W., Cooper S., Brooke J.M., Knowles J.D. (2016). Fifty years of pulsar candidate selection: From simple filters to a new principled real-time classification approach. Mon. Not. R. Astron. Soc..

[23] Vafaei Sadr A., Bassett B.A., Oozeer N., Fantaye Y., Finlay C. (2020). Deep learning improves identification of radio frequency interference. Mon. Not. R. Astron. Soc..

[24] Sadhu A. (2022). Pulsar Star Detection: A Comparative Analysis of Classification Algorithms using SMOTE. Int. J. Comput. Inf. Technol..

[25] Bhat S.S., Prabu T., Stappers B., Ghalame A., Saha S., Sudarshan T.B., Hosenie Z. (2023). Investigation of a Machine learning methodology for the SKA pulsar search pipeline. J. Astrophys. Astron..

[26] Wang Y., Pan Z., Zheng J., Qian L., Li M. (2019). A hybrid ensemble method for pulsar candidate classification. Astrophys. Space Sci..

[27] Guo P., Duan F., Wang P., Yao Y., Yin Q., Zhang L. (2019). Pulsar candidate classification using generative adversary networks. Mon. Not. R. Astron. Soc..

[28] Song J.R. (2023). The effectiveness of different machine learning algorithms in classifying pulsar stars and the impact of data preparation. J. Phys. Conf. Ser..

[29] Ding X., Zhang Y., Ge Y., Zhao S., Song L., Yue X., Shan Y. UniRepLKNet: A Universal Perception Large-Kernel ConvNet for Audio Video Point Cloud Time-Series and Image Recognition. Proceedings of the IEEE/CVF Conference on Computer Vision and Pattern Recognition.

[30] Slabbert D., Lourens M., Petruccione F. (2024). Pulsar classification: Comparing quantum convolutional neural networks and quantum support vector machines. Quantum Mach. Intell..

[31] Zhang C.J., Shang Z.H., Chen W.M., Miao X.H. (2020). A review of research on pulsar candidate recognition based on machine learning. Procedia Comput. Sci..

[32] Ji L., Liu N. (2025). Global-local transformer for aerial image semantic segmentation. Proceedings of the Sixteenth International Conference on Graphics and Image Processing (ICGIP 2024).

[33] Lian X., Huang X., Gao C., Ma G., Wu Y., Gong Y., Li J. (2023). A Multiscale Local–Global Feature Fusion Method for SAR Image Classification with Bayesian Hyperparameter Optimization Algorithm. Appl. Sci..

[34] Dosovitskiy A. (2020). An image is worth 16 × 16 words: Transformers for image recognition at scale. arXiv.

[35] Bao F., Nie S., Xue K., Cao Y., Li C., Su H., Zhu J. All are worth words: A vit backbone for diffusion models. Proceedings of the IEEE/CVF Conference on Computer Vision and Pattern Recognition.

[36] Xia C., Wang X., Lv F., Hao X., Shi Y. Vit-comer: Vision transformer with convolutional multi-scale feature interaction for dense predictions. Proceedings of the IEEE/CVF Conference on Computer Vision and Pattern Recognition.

[37] Yuan L., Chen Y., Wang T., Yu W., Shi Y., Jiang Z.H., Yan S. Tokens-to-token vit: Training vision transformers from scratch on imagenet. Proceedings of the IEEE/CVF International Conference on Computer Vision.

[38] Dong X., Bao J., Chen D., Zhang W., Yu N., Yuan L., Guo B. Cswin transformer: A general vision transformer backbone with cross-shaped windows. Proceedings of the IEEE/CVF Conference on Computer Vision and Pattern Recognition.

[39] Wang W., Xie E., Li X., Fan D.P., Song K., Liang D., Shao L. (2022). Pvt v2: Improved baselines with pyramid vision transformer. Comput. Vis. Media.

[40] Brown T., Mann B., Ryder N., Subbiah M., Kaplan J.D., Dhariwal P., Amodei D. (2020). Language models are few-shot learners. Adv. Neural Inf. Process. Syst..

[41] Hendricks L.A., Mellor J., Schneider R., Alayrac J.B., Nematzadeh A. (2021). Decoupling the role of data, attention, and losses in multimodal transformers. Trans. Assoc. Comput. Linguist..

[42] Ding X., Zhang X., Han J., Ding G. Scaling up your kernels to 31 × 31: Revisiting large kernel design in cnns. Proceedings of the IEEE/CVF Conference on Computer Vision and Pattern Recognition.

[43] Mehta P., Sagar A., Kumari S. (2024). Domain Generalized Recaptured Screen Image Identification Using SWIN Transformer. arXiv.

[44] Shi L., Chen Y., Liu M., Guo F. DuST: Dual Swin Transformer for Multi-modal Video and Time-Series Modeling. Proceedings of the IEEE/CVF Conference on Computer Vision and Pattern Recognition.

[45] Liu Z., Lin Y., Cao Y., Hu H., Wei Y., Zhang Z., Guo B. Swin transformer: Hierarchical vision transformer using shifted windows. Proceedings of the IEEE/CVF International Conference on Computer Vision.

[46] Zhu X., Wu Y., Hu H., Zhuang X., Yao J., Ou D., Xu D. (2022). Medical lesion segmentation by combining multimodal images with modality weighted UNet. Med. Phys..

[47] Prakash A., Chitta K., Geiger A. Multi-modal fusion transformer for end-to-end autonomous driving. Proceedings of the IEEE/CVF Conference on Computer Vision and Pattern Recognition.

[48] Liu C., Zou W., Hu Z., Li H., Sui X., Ma X., Guo N. (2024). Bearing Health State Detection Based on Informer and CNN+ Swin Transformer. Machines.

[49] Zhou H.Y., Yu Y., Wang C., Zhang S., Gao Y., Pan J., Li W. (2023). A transformer-based representation-learning model with unified processing of multimodal input for clinical diagnostics. Nat. Biomed. Eng..

[50] Akbari H., Yuan L., Qian R., Chuang W.H., Chang S.F., Cui Y., Gong B. (2021). Vatt: Transformers for multimodal self-supervised learning from raw video, audio and text. Adv. Neural Inf. Process. Syst..

[51] Wen Y., Zhang K., Li Z., Qiao Y. (2019). A comprehensive study on center loss for deep face recognition. Int. J. Comput. Vis..

[52] Han C., Pan P., Zheng A., Tang J. (2021). Cross-modality person re-identification based on heterogeneous center loss and non-local features. Entropy.

[53] Wen Y., Zhang K., Li Z., Qiao Y. (2016). A discriminative feature learning approach for deep face recognition. Proceedings of the Computer vision—ECCV 2016: 14th European Conference.

[54] Wen S., Liu W., Yang Y., Zhou P., Guo Z., Yan Z., Huang T. (2020). Multilabel image classification via feature/label co-projection. IEEE Trans. Syst. Man Cybern. Syst..

[55] Dai Y., Li X., Liu J., Tong Z., Duan L.Y. Generalizable person re-identification with relevance-aware mixture of experts. Proceedings of the IEEE/CVF Conference on Computer Vision and Pattern Recognition.

[56] Jiang M., Zhang X., Yu Y., Bai Z., Zheng Z., Wang Z., Yang Y. Robust vehicle re-identification via rigid structure prior. Proceedings of the IEEE/CVF Conference on Computer Vision and Pattern Recognition.

[57] Tan L., Zhang Y., Shen S., Wang Y., Dai P., Lin X., Ji R. (2023). Exploring invariant representation for visible-infrared person re-identification. arXiv.

